# Molecular Diversity by Olefin Cross-Metathesis on Solid Support. Generation of Libraries of Biologically Promising β-Lactam Derivatives

**DOI:** 10.3390/molecules23051193

**Published:** 2018-05-16

**Authors:** Luciana Méndez, Andrés A. Poeylaut-Palena, Ernesto G. Mata

**Affiliations:** Instituto de Química Rosario, Facultad de Ciencias Bioquímicas y Farmacéuticas, Universidad Nacional de Rosario-CONICET, Suipacha 531, Rosario S2002LRK, Argentina; mendez@iquir-conicet.gov.ar (L.M.); andrespoeylaut@gmail.com (A.A.P.-P.)

**Keywords:** diversity oriented synthesis, solid-phase organic synthesis, β-lactam derivatives, olefin metathesis, cholesterol absorption inhibitors

## Abstract

The application of the reagent-based diversification strategy for generation of libraries of biologically promising β-lactam derivatives is described. Key features are the versatility of the linker used and the cross-metathesis functionalization at the cleavage step. From an immobilized primary library, diversity was expanded by applying different cleavage conditions, leading to a series of cholesterol absorption inhibitor analogues together with interesting hybrid compounds through incorporation of a chalcone moiety.

## 1. Introduction

The β-lactam skeleton is an active acylating agent with numerous applications in clinical therapy [1]. Besides the well-known use as antibiotics [2], β-lactam ring has inhibitory effects on prostate specific antigen [3,4], thrombin [5], human cytomegalovirus protein [6], human leukocyte elastase [7], cysteine protease [8,9], and human fatty acid amide hydrolase [10], as well as anticancer properties [11,12,13,14,15] and neuroprotective action [16,17]. A new area in β-lactam-based drugs has been established by development of β-lactam derivatives with strong cholesterol absorption inhibitor reducing LDL concentration [18,19,20,21]. An example is the commercial drug ezetimibe, which is one of the most prescribed drugs in the US. Recently, simvastatin has been shown to provide an incremental benefit on reducing cardiovascular events in acute coronary syndrome patients, when co-administered with ezetimibe [22]. Furthermore, β-lactams have also been considered as peptidomimetic species for mimicking certain properties of proteins and, from a synthetic point of view, they are key synthons for the preparation of various heterocyclic compounds of biological importance [23,24,25].

**Scheme 1:**
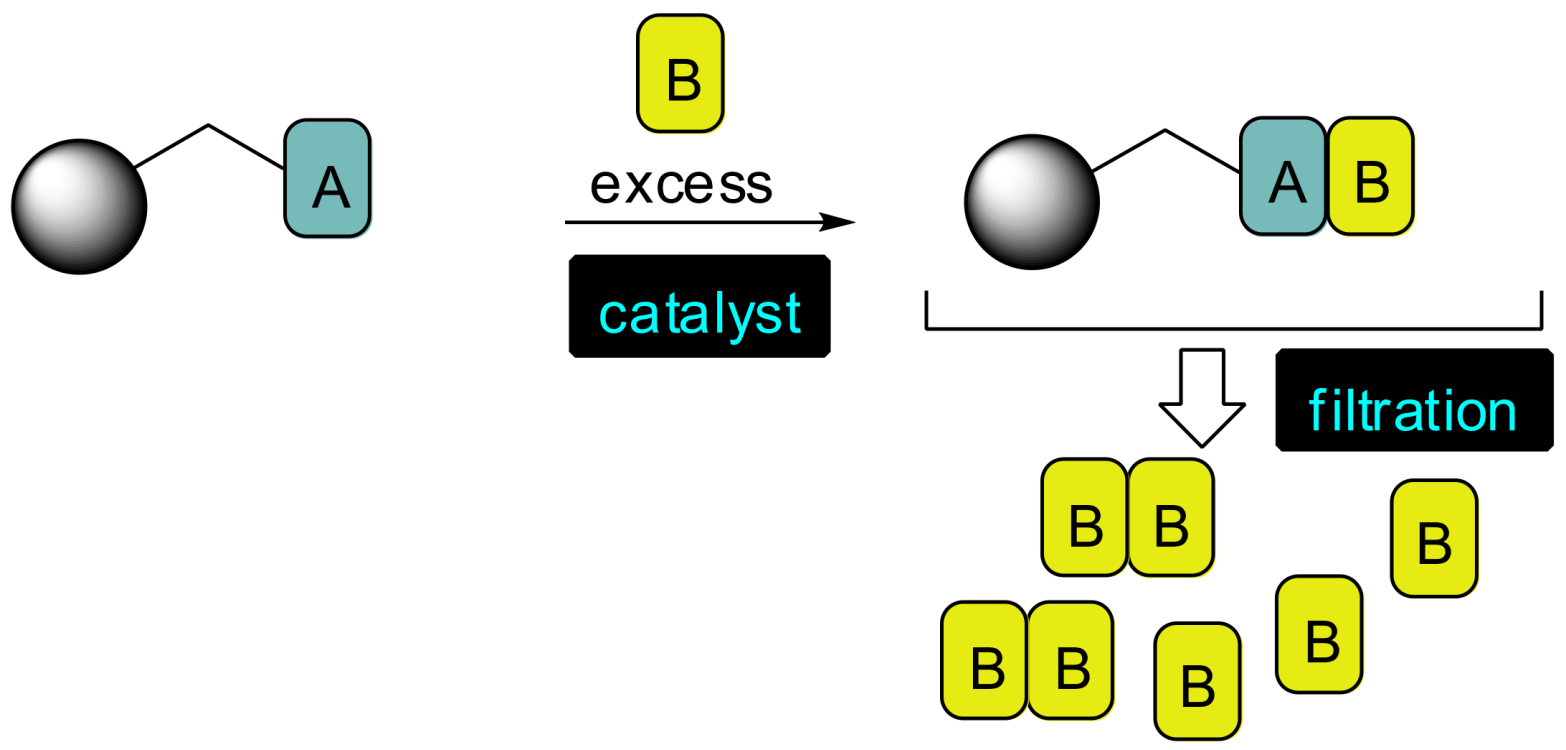
Metal-catalyzed solid-phase synthesis.

In drug discovery related organic synthesis, one of the tools for a rapid and efficient construction of diversity-based small molecules is parallel solid-phase synthesis. Particularly, solid-phase chemistry has recently aroused interest in metal-catalyzed cross-coupling reactions since undesirable soluble homodimers can be washed away during purification providing chemoselectivity, while immobilization of one of the substrates makes its homodimerization a less favorable process due to site isolation (Scheme 1) [26]. In this regard, we and others have recognized the usefulness of solid-supported olefin cross-metathesis for generation of biologically relevant molecules [27,28,29,30,31], including a comprehensive study to understand the process [32,33]. From the viewpoint of green chemistry, using solid-phase synthetic sequences allows a significant reduction in solvent waste, since purification is performed by phase separation, avoiding chromatographic isolation of products which requires a large consumption of organic solvents [34].

In the search of diversity, a bunch of very ingenious strategies have been developed, among which diversity-oriented synthesis (DOS) is the most widespread [35,36,37]. According to DOS, skeletal diversity can be basically achieved by two main approaches: one known as “substrate-based diversification” where the same reagent generates different structures by reaction with selected substrates; and “reagent-based diversification” in which different products are obtained when the same substrate is subjected to different reaction conditions [38].

**Scheme 2:**
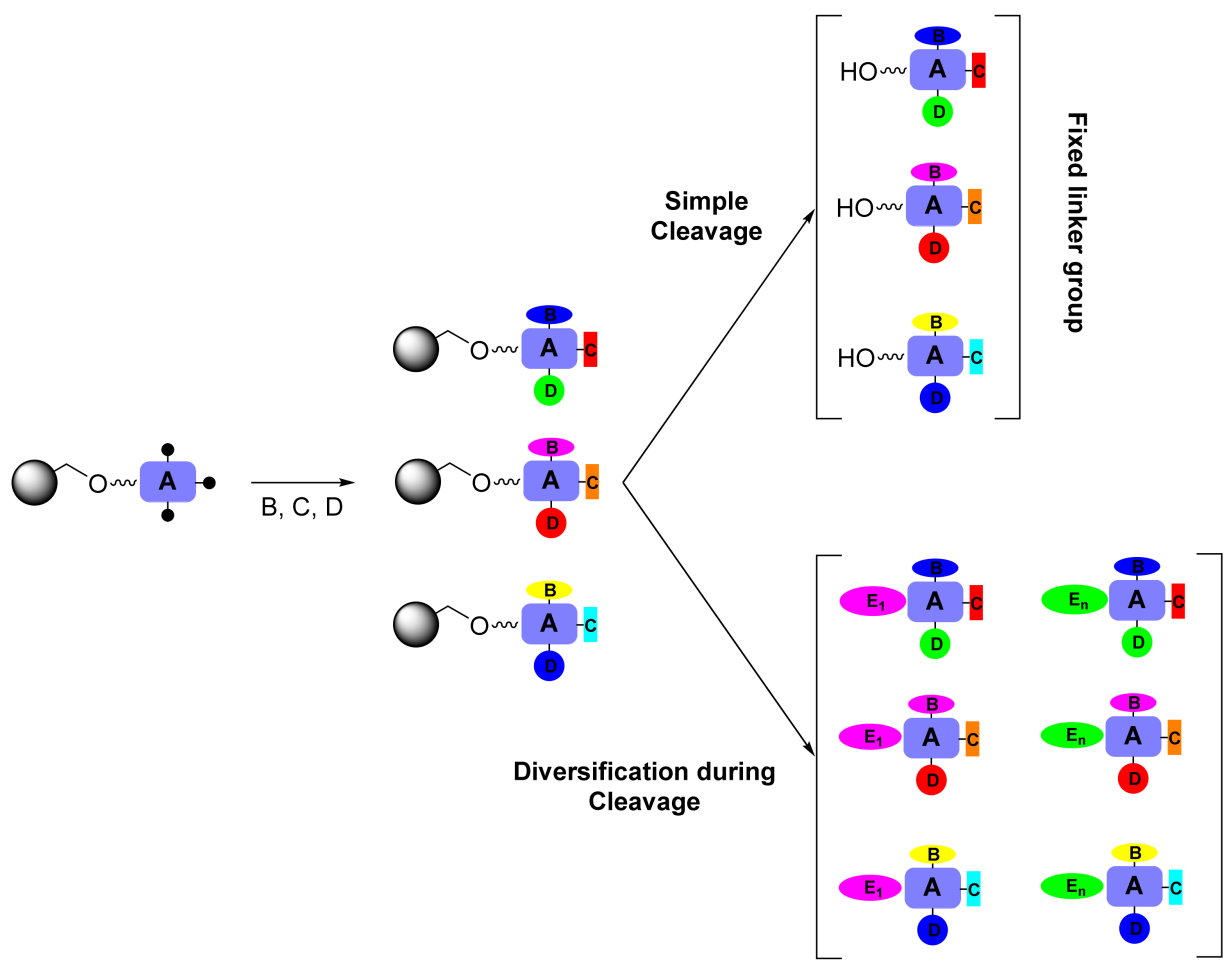
Reagent-based diversification approach in solid-phase synthesis.

Generally, the point of attachment to the resin in solid-phase organic synthesis (SPOS) limits the possibilities of diversity generation. In previous work, we have developed a series of β-lactam compounds linked to the resin at position 1 or 3 of the ring. Only the substituents on the two remaining positions could be combined [27,39]. In order to maximize structural variation from a library of compounds, we have studied a strategy based on a linker, which ensures release by different reaction conditions to obtain diverse products using a DOS reagent-based diversification approach (Scheme 2).

We have shown that high molecular diversity in β-lactam scaffolds can be obtained using a linker cleavable by two orthogonal conditions: one by treatment with 10% TFA, and the other using a set of olefin cross-metathesis reactions. While Seeberger developed olefin linkers for solid-phase synthesis of glycosides [40], we decided to introduce a new twist using them to generate diversity during cleavage step. Thus, a series of cholesterol absorption inhibitor analogues, together with interesting β-lactam-chalcone hybrid compounds, have been generated. Our results are summarized in this extended paper [41].

## 2. Results and Discussion

**Scheme 3:**
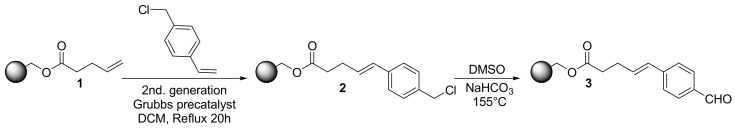
Synthesis of versatile linker **3**.

**Figure 1:**
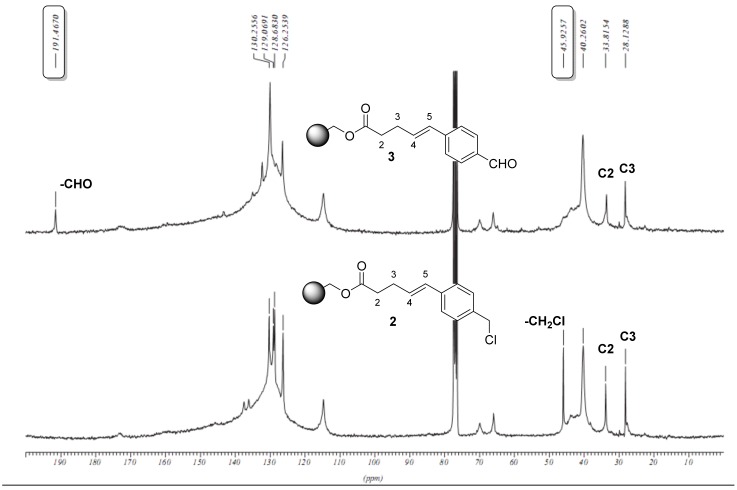
^13^C gel-phase NMR of compounds **2** and **3**.

Synthesis started with immobilization of 4-pentenoic acid to Wang resin by standard coupling conditions, to afford the corresponding resin **1** which, in turn, was the substrate for a solid-supported olefin cross-metathesis by reaction with 1-(chloromethyl)-4-vinylbenzene in the presence of second generation Grubbs precatalyst, according to the methodology reported by us (Scheme 3) [32]. Then, oxidation of the supported benzyl chloride **2** with DMSO/NaHCO_3_ at 155 °C [42] yielded the aldehyde **3**, which was characterized by ^13^C gel-phase NMR (Figure 1). Aldehyde **3** was used as substrate for the synthesis of libraries of β-lactam derivatives.

### 2.1. Primary β-Lactam Library

**Scheme 4:**
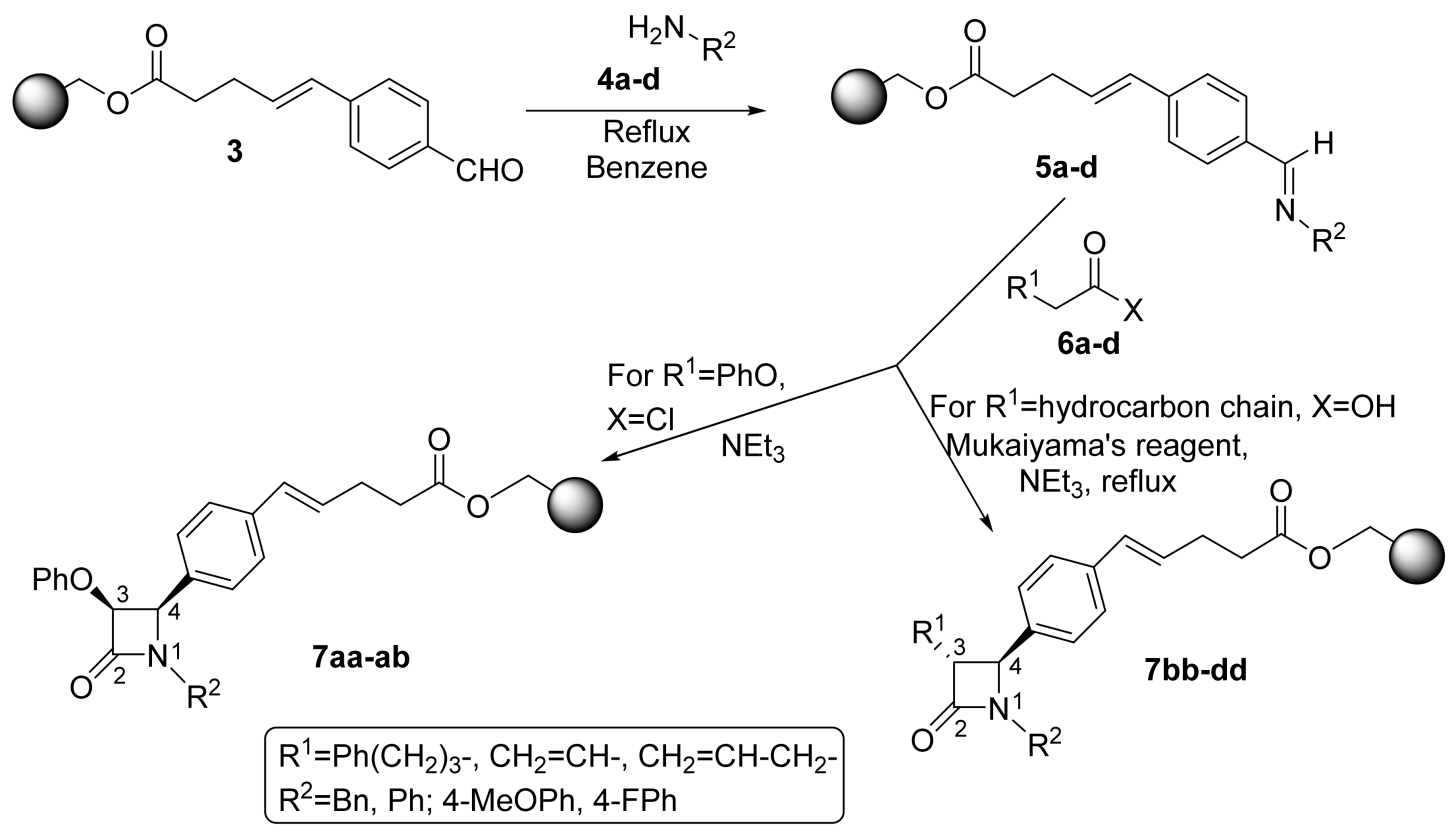
Primary immobilized β-lactam library.

For the solid-phase version of the classical Staudinger reaction between imines and ketenes [43,44,45], we first developed the synthesis of a set of immobilized imines **5a**–**d** by treatment of the aldehyde with different amines **4a**–**d** in refluxing benzene using a Dean–Stark trap (Scheme 4).

For the in situ generation of ketenes, we found that stable acid chlorides such as phenoxyacetyl chloride were ideal, however, when aliphatic carboxylic acids derivatives were required, the best choice was the activation of the free acid by Mukaiyama’s reagent. Thus, immobilized imines **5a**–**d** were treated with either acid chloride or carboxylic acids (**6a**–**d**) and Mukaiyama’s reagent to obtain the primary β-lactam library (**7aa**–**dd**) (Scheme 4).

**Figure 2:**
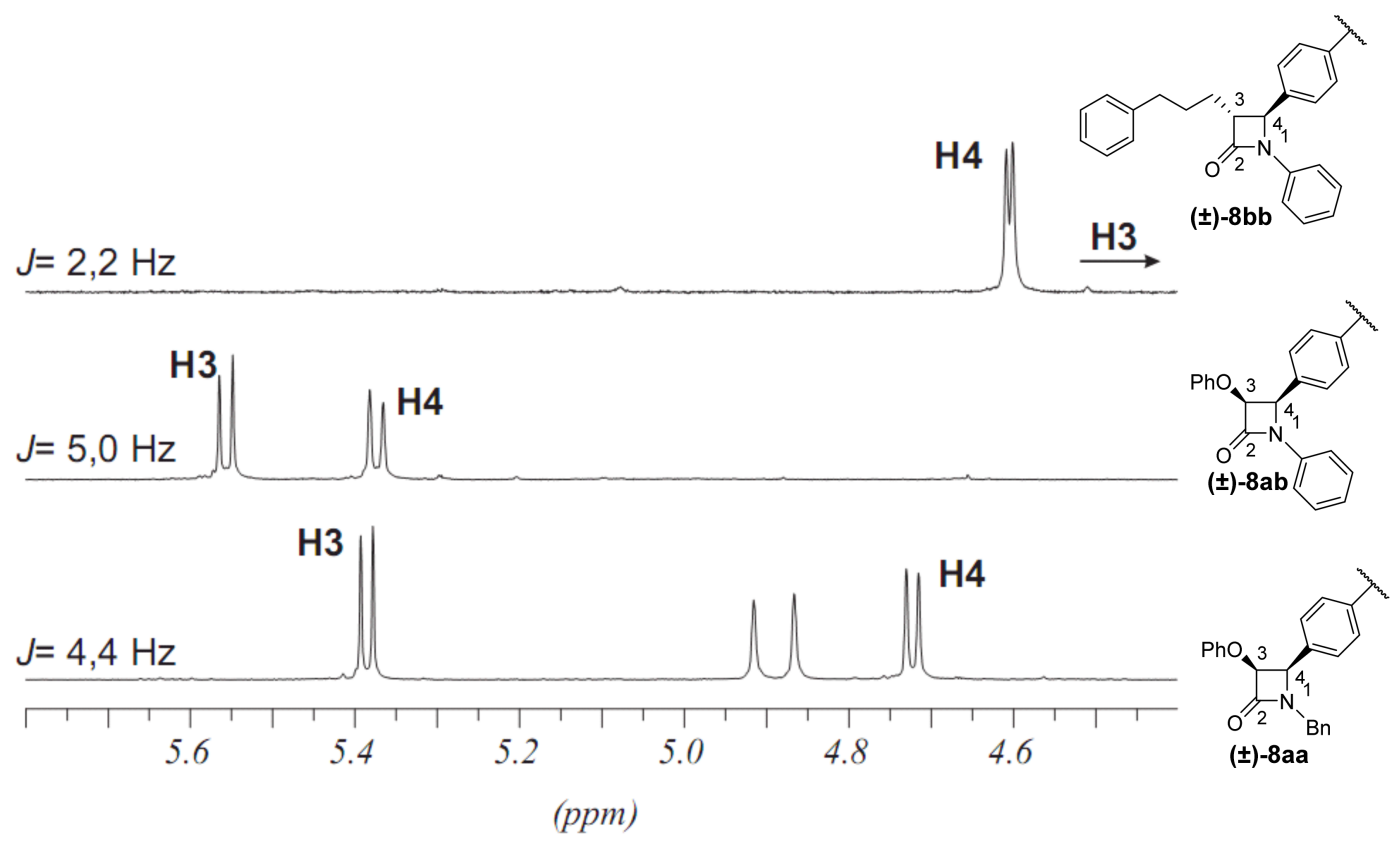
Comparative ^1^H-NMR spectra of 3,4-*cis*- and *trans*-substituted β-lactams.

The relative configuration of C-3 and C-4 substituents was determined taking the coupling constant between H-3 and H-4 nucleus in ^1^H-NMR experiments after trifluoroacetic acid (TFA) cleavage into account. Coupling constants greater than 4 Hz were indicative of a *cis* configuration [46], so β-lactams **8aa** and **8ab** had a *cis* configuration while **8bb** had a *trans* configuration (Figure 2 and Table 1).

During Staudinger reaction, the diastereoselectivity for *cis* or *trans* configuration of the resulting β-lactams was governed by substituents on the starting building blocks (imine and ketene) [47,48]. Ketenes that were activated by heteroatoms had strong preference for formation of *cis* products. This was the case for β-lactams **8aa** (*J* = 5.0 Hz) and **8ab** (*J* = 4.4 Hz) that have been constructed from ketenes derived from dehydrohalogenation of phenoxyacetyl chloride. On the other hand, β-lactam **8bb**, which was synthesized from an alkyl-ketene derivative obtained from 5-phenylvaleric acid, gave a *trans* configuration (*J* = 2.2 Hz). The presence of a heteroatom tethered to the α-position of the ketene and tended to form the *cis* products by conrotatory ring closure of the zwitterionic intermediate, formed through a nucleophilic attack of imine nitrogen on the electrophilic carbonyl carbon of the ketene [47]. Conversely, thermodynamically more stable *trans*-β-lactam **8bb** was generated by isomerization of the zwitterion, when a hydrocarbon chain was tethered to the ketene.

**Figure 3:**
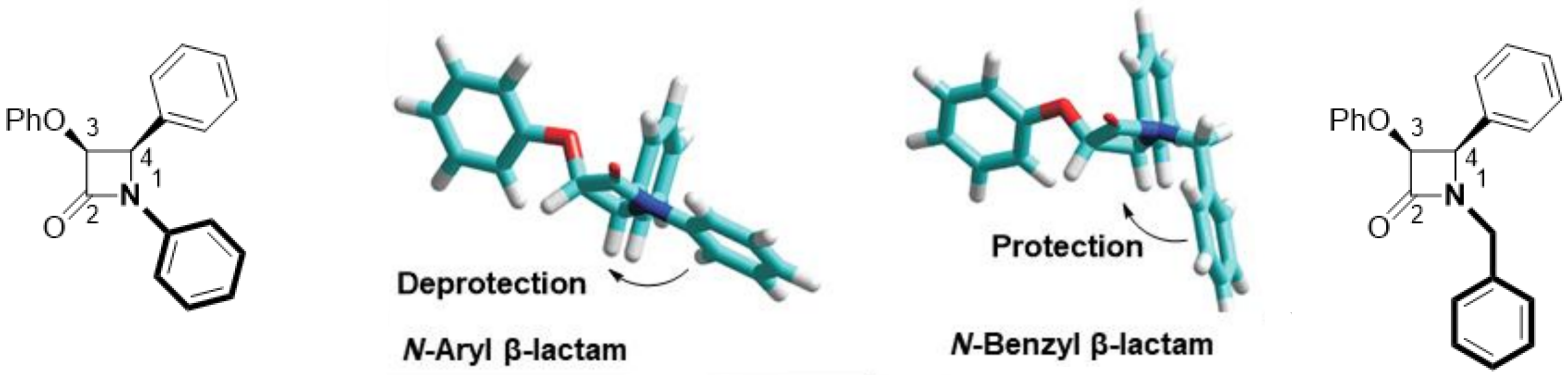
AM1 geometric optimization of *N*-aryl and *N*-benzyl β-lactams.

^1^H-NMR signals corresponding to protons H-3 and H-4 showed a deshielding effect when substituent of position 1 changed from *N*-benzyl to *N*-aryl, respectively (compare **8aa** and **8ab** in Figure 2). Geometric optimization employing a semi-empirical method such as AM1 [49] indicated a radical change in the arrangement of the aromatic ring at position 1 (Figure 3). In the *N*-aryl β-lactam, H-3 and H-4 were in the same plane of the N-1 aromatic ring, while in *N*-benzyl β-lactam these protons were near the axis of the ring. Due to anisotropic effect, protons H-3 and H-4 in the *N*-aryl-β-lactam were in a deshielding area, whereas in *N*-benzyl-β-lactam those protons were in a shielding environment. The absence of an oxygen atom directly linked to position 3 of the β-lactam ring in **8bb**, leading to a drastic shift of the signal to higher fields.

**Figure 4:**
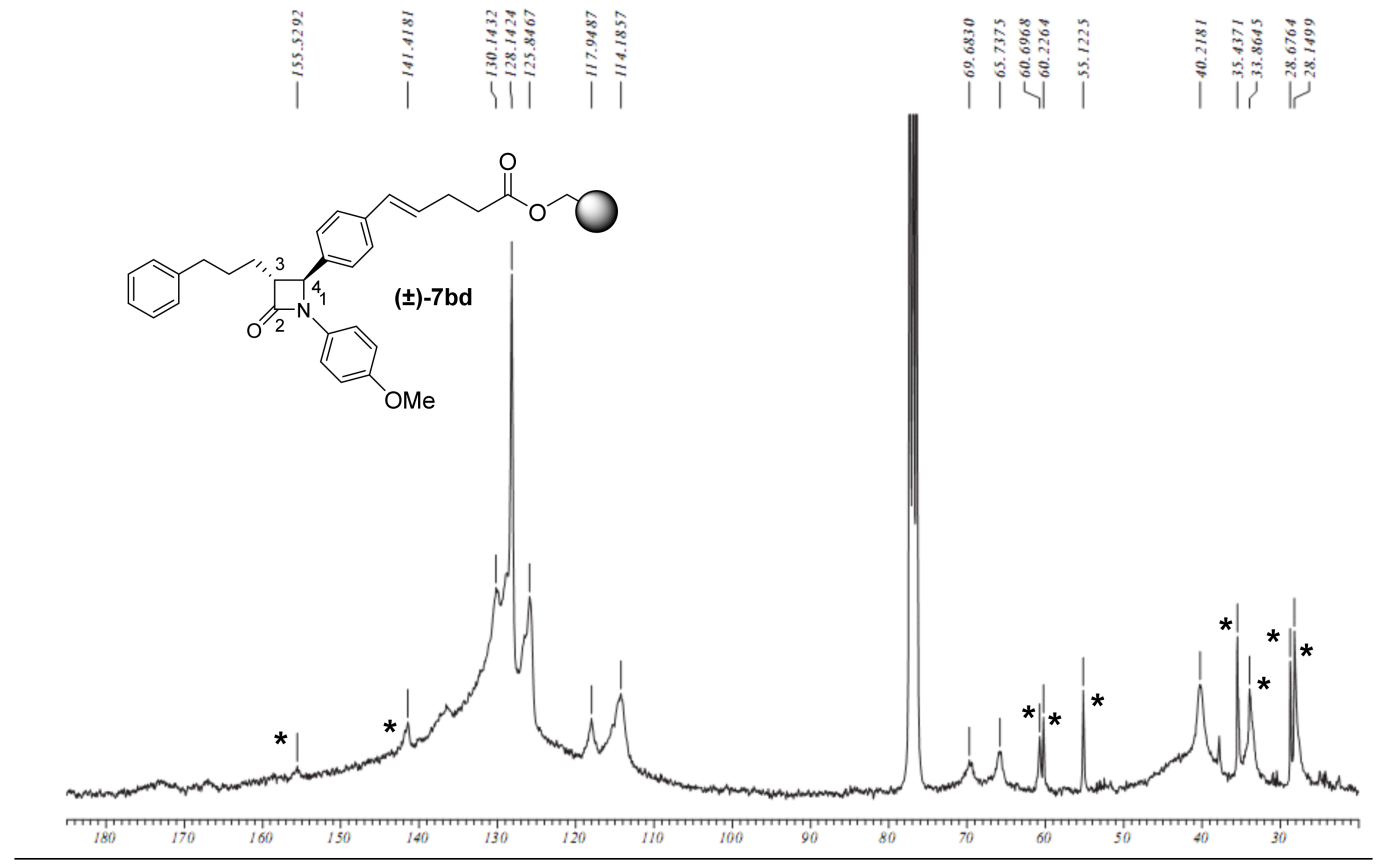
^13^C gel-phase NMR of immobilized β-lactam **7bd**.

Generation of this primary library was monitored by IR and ^13^C gel-phase NMR. A representative example of ^13^C gel-phase NMR of resin **7bd** is shown in Figure 4. Signals assigned to the immobilized β-lactam are marked (*). The most representative signals were 55.1 ppm (MeO-), and 60.7/60.2 ppm corresponding to C-3 and C-4 of the β-lactam ring.

### 2.2. Secondary β-Lactam Libraries

Taking advantage of the multiple possibilities of cleavage, a series of secondary libraries was generated. Thus, TFA treatment of the primary library, followed by diazomethane methylation gave an eleven compounds’ secondary “TFA” library (Table 1).

**Figure 5:**
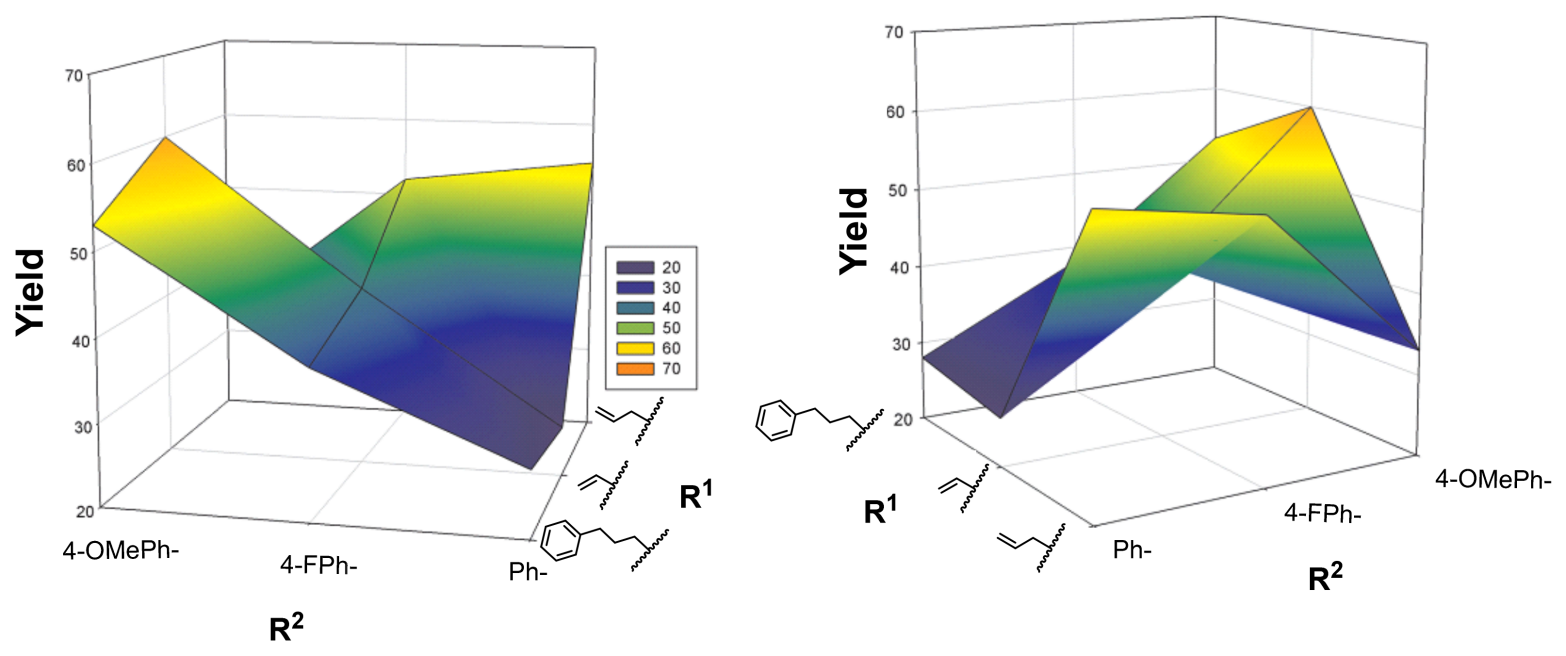
Yields of R^1^ and R^2^ β-lactam substituents.

The efficiency of the solid-phase synthetic strategy has proven to be excellent. Yields ranged from 26% to 60% for five synthetic steps. Analyzing the reaction outcome of the obtained *trans*-β-lactams (entries 3–11), no clear tendency could be observed. While yields increase from R^2^ = Ph to R^2^ = 4–FPh and 4–MeOPh, in the cases of R^1^ = phenylpropyl and vinyl, this tendency was reverted in case of R^1^ = allyl (Figure 5).

In order to increase diversity during cleavage step, we studied the olefin cross-metathesis on the immobilized β-lactams. Thus, a new secondary library was created when β-lactams **7bb**–**bd**, bearing a 3-phenylpropyl group at C-3 position, reacted with different olefins to afford structural diversification at C4 position (Table 2). This secondary library, called “Ru1”, was present in six different alternative β-lactams (**10bba**–**bdc**) obtained by reaction of the olefins in presence of second generation Grubbs precatalyst. In accordance with our previous results in the area of olefin cross-metathesis [32], *trans*-crotonic acid (entries 1 and 2), a type I olefin [50] and allylbenzene (entries 3 and 4), a type II olefin, performed the cleavage process with high efficiency, while 2-bromostyrene (entries 5 and 6), a type IV olefin, provided a more poor yield.

Hybrid structures, obtained from combining at least two biologically significant moieties, have emerged as a novel approach in finding new chemical entities [51,52,53]. The molecular hybridization strategy has proven to be helpful in many aspects related to drug discovery, such as overcoming drug-resistance problems or improving active transport mechanisms. In particular, β-lactam-based hybrids have recently acquired importance owing to the fact that many of them exhibit very promising biological activity [54,55,56,57,58,59]. In order to further increase diversity and also obtain biologically interesting β-lactam-based hybrids, metathesis conditions, previously developed for the synthesis of chalcones, were applied for the cleavage step (Table 3) [60]. In this secondary library, called “Ru2”, cleavage conditions were performed by reaction of β-lactams **7bc** and **7bd** with non-immobilized α,β-unsaturated ketones, being the most efficient precatalyst of Hoveyda-Grubbs carbene ruthenium complex [61,62,63]. Thus, immobilized β-lactams **7bc**–**bd** were treated with substituted vinyl phenyl ketones **9d**–**f** in the presence of Hoveyda-Grubbs precatalyst to yield the soluble compounds **10bcd**–**bdg** which combined two recognized pharmacophoric moieties such as azetidinone and chalcone in one molecule.

**Figure 6:**
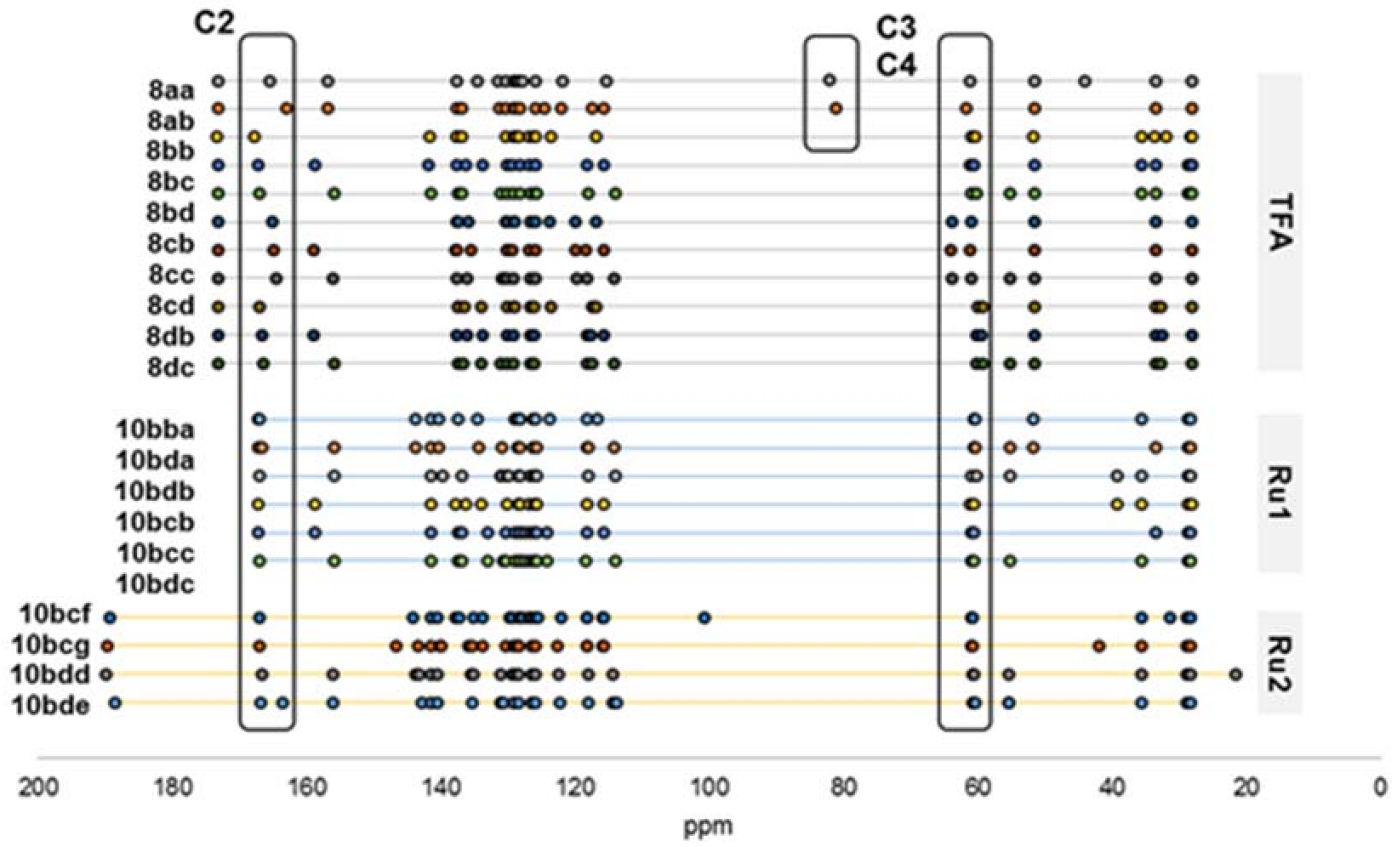
Comparative chart of the ^13^C-NMR signals of the library components.

Through the analysis of the ^13^C-NMR spectra of the components of obtained libraries, a clear pattern could be determined (Figure 6). Signals corresponding to substituents of all positions are slightly affected by remaining substituents. For instance, 4-methoxy-phenyl substituent at position 1 in azetidinones **8bd**, **8cd**, **8dd**, **10bda**, **10bdb**, **10bdc**, **10bdd** and **10bde** showed similar chemical shifts regardless the remaining substituents of β-lactam. Signals of the three carbons of the β-lactam ring (C-2, C-3 and C-4) could clearly be identified, having only a remarkable and expected low-field chemical shift of C-3 in case of 3-phenoxy-*cis*-β-lactams (**8aa** and **8ab**). Although there was the possibility that other signals could interfere, the patterns shown in Figure 6 were useful for a rapid detection of β-lactam ring containing compounds obtained by high throughput parallel synthesis.

## 3. Materials and Methods

### 3.1. General Information

Chemical reagents were purchased from commercial suppliers and used without further purification, unless otherwise noted. Solvents were analytical grade or were purified by standard procedures prior to use. Reactions requiring inert atmosphere were carried out under high-purity dry nitrogen atmosphere. Solvents from these reactions were transferred under high-purity dry nitrogen using syringes. All reactions were monitored by thin layer chromatography (Merck, Darmstadt, Germany) performed on silica gel 60 F254 pre-coated aluminum sheets, visualized by a 254 nm UV lamp, and stained with an ethanolic solution of 4-anisaldehyde. Column flash chromatography (Merck, Darmstadt, Germany) was performed using silica gel 60 (230–400 mesh). The purity criteria were (i) the appearance of a single spot by thin layer chromatography (ii) the presence of the corresponding signals in ^1^H and ^13^C-NMR and (iii) the range of melting points in case of solid samples. Molecular modeling was performed with HyperChem v8.03 (Hypercube, Gainesville, FL, USA) using the AM1 method

Solid-phase reactions were carried out in polypropylene cartridges equipped with a frit (Supelco, Bellefonte, PA, USA), unless reflux conditions were required. In that case, standard glassware was used. All solid-phase reaction mixtures were stirred at slowest rate. Compounds **8bb**–**8dd** and **10bba**–**10bdc** have been previously reported [41].

### 3.2. Instrumental and Physical Data

^1^H-NMR spectra were recorded in a Bruker Avance spectrometer (Bruker Analytik GmbH, Karlsruhe, Germany) at 300 MHz in CDCl_3_ with tetramethylsilane (TMS) as internal standard (0 ppm). ^13^C-NMR spectra were recorded on the same apparatus at 75 MHz with CDCl_3_ as solvent and reference (76.9 ppm). Chemical shifts (δ) were reported in ppm upfield from TMS and coupling constants (*J*) were expressed in Hertz. The following abbreviations were used to indicate multiplicities: s = singlet, d = doublet, t = triplet, q = quartet, m = multiplet, and bs = broad singlet. For preparation of the samples for ^13^C gel-phase NMR, 50–80 mg of resin was placed in a standard NMR tube and 0.5 mL of CDCl_3_ was slowly added in order to obtain a gel, which was homogenized by sonication. Spectra were run according to the literature [64,65].

Infrared spectra were recorded on a Shimadzu FT-IR spectrometer model 8101 (Shimadzu, Tokyo, Japan). The resin samples were measured as dispersions in KBr discs, made by compression of a mixture finely powdered in an agate mortar. Approximately 3 mg sample was used in 100 mg KBr.

### 3.3. Synthetic Procedures

*General procedure for the synthesis of 3-phenoxy-β-lactam derivatives* (**8aa**–**ab**) *and β-lactam-chalcone hybrids* (**10bdd**–**bcg**): a mixture of 4-pentenoic acid (420 μL, 4.15 mmol, 5.0 eq.) in anhydrous DMF (10 mL) and DCC (830 μL, 4.15 mmol, 5.0 eq.) was stirred for 30 min at room temperature and transferred via cannula to Wang resin (750.0 mg, 1.1 mmol/g, 0.83 mmol), which was previously rinsed with anhydrous DMF (5.0 mL). After adding DMAP (100.0 mg, 0.83 mmol, 1.0 eq.), the reaction mixture was stirred for 16 h at room temperature. Then, resin was washed with DMF (3 × 10 mL), 1% AcOH in AcOEt (3 × 10 mL), AcOEt (3 × 10 mL), MeOH (3 × 10 mL) and CH_2_Cl_2_ (3 × 10 mL), and dried in vacuo. The immobilized pentenoate (**1**) (820 mg, 0.83 mmol) was suspended in anhydrous CH_2_Cl_2_ (20 mL) and 4-vinylbenzyl chloride (590 μL, 4.15 mmol, 5.0 eq.) was added via syringe under a nitrogen atmosphere. After that, Grubbs’ second generation pre-catalyst (35.0 mg, 41.5 μmol, 5 mol %) was added and the reaction was refluxed for 20 h. The mixture was then filtered, washed with CH_2_Cl_2_ (3 × 10 mL), MeOH (3 × 10 mL), CH_2_Cl_2_ (1 × 10 mL), and dried under high vacuum. The procedure was repeated once to ensure complete reaction. In the next step, NaHCO_3_ (68.5 mg, 2.2 eq.) in DMSO (25.0 mL) was added to the immobilized benzyl chloride (**2**) (915.0 mg, 0.37 mmol) and the mixture was heated to 155 °C for 6 h. After that, the suspension was filtered, washed with DMSO (3 × 10 mL), CH_2_Cl_2_ (3 × 10 mL), MeOH (3 × 10 mL), CH_2_Cl_2_ (1 × 10 mL) and dried in vacuo to obtain the Wang resin-linked aldehyde **3**. In the next step, the immobilized aldehyde **3** (121.2 mg; 0.13 mmol) was placed in a round-bottom flask and suspended in anhydrous benzene (20 mL) and the corresponding amine (10 equiv.) was added. A Dean-Stark trap was then fitted, filled with molecular sieves 4 Å and the suspension was heated in reflux for 14 h. After that, the resin was filtered, washed with benzene (3 × 4 mL), DCM (3 × 4 mL), MeOH (3 × 4 mL) and CH_2_Cl_2_ (1 × 4 mL), and dried in vacuo to obtain the resin-bound imine **5**.

*For 3-phenoxy-β-lactam derivatives* (**8aa**–**ab**): to an aliquot of immobilized imine **5a**–**b** (0.11 mmoles)**,** triethylamine (0.30 mL, 2.2 mmoles, 20 eq.) and phenoxyacetyl chloride (0.23 mL, 1.65 mmoles, 15 equiv.) was added at 0 °C. The suspension was stirred for 16 h at room temperature. After filtration, the resin was washed with CH_2_Cl_2_ (3 × 4 mL), AcOEt (3 × 4 mL), MeOH (3 × 4 mL), CH_2_Cl_2_ (1 × 4 mL), and dried under high vacuum. Then, a 10% solution of TFA in CH_2_Cl_2_ (3 mL) was added to the polymer-bound *β*-lactam **7aa**–**ab**. The reaction mixture was stirred for 50 min at room temperature, filtered, and washed with CH_2_Cl_2_ (3 mL). The filtrate was evaporated under reduced pressure. Esterification with diazomethane afforded the crude product that was then purified by column chromatography (hexane-AcOEt).

*For β-lactam-chalcone hybrids* (**10bdd**–**bcg**): 5-phenylvaleric acid (49 mg, 0.28 mmoles, 2.5 equiv.) and triethylamine (90 µL, 0.66 mmoles, 6 equiv.) were dissolved in anhydrous chloroform (3 mL) and added to a suspension of immobilized imine **5b**–**d** (0.11 mmoles) in anhydrous chloroform (1.5 mL) under nitrogen atmosphere. After one minute, 2-chloro-1-methylpyridinium iodide (Mukaiyama’s reagent, 84.3 mg, 0.33 mmoles, 3 equiv.) was added and the suspension was stirred at room temperature for 24 h. Then, the reaction mixture was filtered and the resin was washed successively with CH_2_Cl_2_ (3 × 4 mL), AcOEt (3 × 4 mL), MeOH (3 × 4 mL), and CH_2_Cl_2_ (1 × 4 mL). Resin **7bc**–**bd** (0.11 mmol) was placed in a 25 mL round-bottom flask, purged with dry nitrogen, suspended in anhydrous toluene (3 mL) and olefin **9d**–**f** (0.55 mmol, 5 eq.) dissolved in anhydrous toluene (3 mL) was added via syringe. After addition of Hoveyda-Grubbs precatalyst (3.4 mg, 5.5 µmol, 5 mol %), the flask was fitted with a reflux condenser with a cannula adapted to allow the elimination of generated ethylene during the reaction. The system was heated to 75 °C for one hour under nitrogen atmosphere. The mixture was filtered and the filtrate was evaporated under reduced pressure to afford the crude product. The solvent was evaporated under reduced pressure and the crude material was purified by flash column chromatography (hexane-AcOEt).

### 3.4. Analytical Data

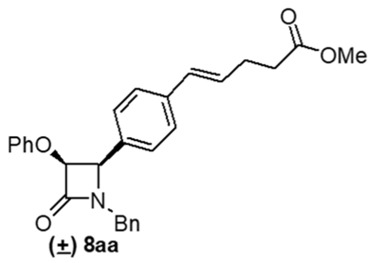



*Methyl (E)-5-(4-(1-benzyl-4-oxo-3-phenoxyazetidin-2-yl)phenyl)pent-4-enoate*: RMN de ^1^H (CDCl_3_, 300 MHz): δ 7.37–7.05 (m, 11H), 6.90–6.82 (m, 1H), 6.77–6.68 (m, 2H), 6.39 (d, *J* = 15.9 Hz, 1H), 6.20 (dt, *J*_1_ = 15.9 Hz, *J*_2_ = 6.6 Hz, 1H), 5.39 (d, *J* = 4.4 Hz, 1H), 4.89 (d, *J* = 14.6 Hz, 1H), 4.72 (d, *J* = 4.4 Hz, 1H), 3.85 (d, *J* = 14.6 Hz, 1H), 3.69 (s, 3H), 2.60–2.40 (m, 4H). RMN de ^13^C (CDCl_3_, 75 MHz): δ 173.2, 165.4, 156.9, 137.6, 134.6, 131.4, 130.3, 129.1, 129.0, 128.8, 128.7, 128.5, 127.8, 125.9, 121.9, 115.4, 82.1, 61.1, 51.5, 44.0, 33.6, 28.1.

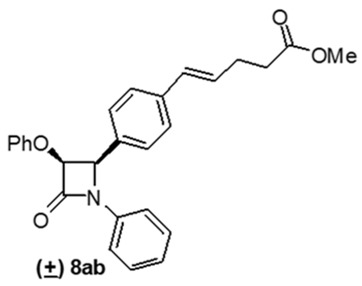



*Methyl (E)-5-(4-(4-oxo-3-phenoxy-1-phenylazetidin-2-yl)phenyl)pent-4-enoate*: RMN de ^1^H (CDCl_3_, 300 MHz): δ 7.40–7.02 (m, 11H), 6.98–6.90 (m, 1H), 6.86–6.75 (m, 2H), 6,37 (d, *J* = 15.9 Hz, 1H), 6.18 (dt, *J*_1_ = 15.9 Hz, *J*_2_ = 6.2 Hz, 1H), 5.56 (d, *J* = 5.0 Hz, 1H), 5.37 (d, *J* = 5.0 Hz, 1H), 3.68 (s, 3H), 2.55–2.40 (m, 4H). RMN de ^13^C (CDCl_3_, 75 MHz): δ 173.2, 163.0, 156.9, 137.7, 136.8, 131.3, 130.3, 129.2, 129.0, 128.2, 126.0, 124.5, 122.1, 117.5, 115.7, 81.2, 61.8, 51.5, 33.6, 28.1.

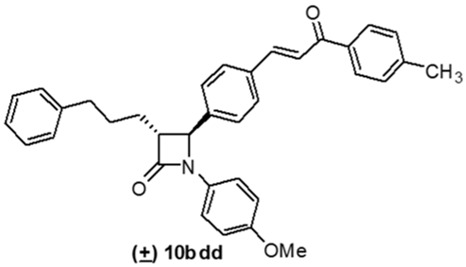



*1-(4-Methoxy-phenyl)-4-[4-(3-oxo-3-p-tolyl-propenyl)-phenyl]-3-(3-phenyl-propyl)-azetidin-2-one*: ^1^H-NMR: 7.92 (d, *J* = 8.1 Hz, 2H), 7.76 (d, *J* = 15.9 Hz, 1H), 7.62 (d, *J* = 8.1 Hz, 2H), 7.51 (d, *J* = 15.9 Hz, 1H), 7.37 (d, *J* = 8.1 Hz, 2H), 7.31–7.14 (m, 7H), 6.78 (d, *J* = 9 Hz, 2H), 4.62 (d, *J* = 2.1 Hz, 1 H), 3.73 (s, 3H), 3.13–3.07 (m, 1H), 2.66 (t, *J* = 7 Hz, 2H), 2.43 (s, 3H), 1.99–185 (m, 4H). ^13^C-NMR: 189.8, 166.7, 156.0, 143.8, 143.3, 141.5, 140.6, 135.5, 135.2, 131.1, 129.3, 129.1, 128.6, 128.4, 126.4, 125.9, 122.5, 118.1, 114.3, 60.8, 60.6, 55.4, 35.7, 28.9, 28.4, 21.6. HRMS calcd. for: C_35_H_33_NNaO_3_^+^; (M + Na^+^, *m*/*z*): 538.23527; found: 538.23673.

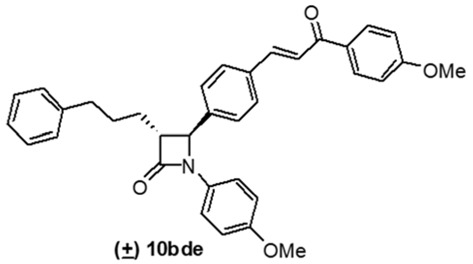



*1-(4-Methoxy-phenyl)-4-{4-[3-(4-methoxy-phenyl)-3-oxo-propenyl]-phenyl}-3-(3-phenyl-propyl)-azetidin-2-one*: ^1^H-NMR: 8.02 (d, *J* = 8.8 Hz, 2H), 7.76 (d, *J* = 15.7 Hz, 1H), 7.63 (d, *J* = 8.2 Hz, 2H), 7.52 (d, *J* = 15.7 Hz, 1H), 7.36 (d, *J* = 8.2 Hz, 2H) 7.30–7.14 (m, 7H), 6.97 (d, *J* = 8.8 Hz, 2H), 6.77 (d, *J* = 9.0 Hz, 2H), 4.62 (d, *J* = 2.1 Hz, 1H), 3.89 (s, 3H), 3.73 (s, 3H), 3.12–2.99 (m, 1H), 2.68–2.63 (m, 2H), 1.98–1.82 (m, 4H). ^13^C-NMR: 188.5, 166.8, 163.5, 156.0, 142.9, 141.5, 140.5, 135.3, 131.1, 130.9, 130.8, 129.1, 128.4, 126.4, 125.9, 122.3, 118.1, 114.3, 113.9, 60.8, 60.5, 55.5, 55.4, 35.7, 28.9, 28.4. HRMS calcd. for C_35_H_33_NNaO_4_^+^ (M + Na^+^, *m/z*): 554.23018; found: 554.22853.

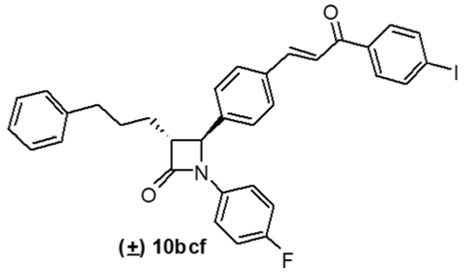



*1-(4-Fluoro-phenyl)-4-{4-[3-(4-iodo-phenyl)-3-oxo-propenyl]-phenyl}-3-(3-phenyl-propyl) azetidin-2-one*: ^1^H-NMR: 7.87 (d, *J* = 8.5 Hz, 2H), 7.78 (d, *J* = 15.6 Hz, 1H), 7.70 (d, *J* = 8.5 Hz, 2H), 7.63 (d, *J* = 8.1 Hz, 2H), 7.44 (d, *J* = 15.6 Hz, 1H), 7.37 (d, *J* = 8.1 Hz, 2H), 7.28–7.14 (m, 7H), 6.93 (m, 2H), 4.63 (d, *J* = 2.1 Hz, 1H), 3.10 (m, 1H), 2.67 (m, 2H), 2.0–1.82 (m 4H). ^13^C-NMR: 189.4, 167.0, 144.2, 141.4, 140.5, 137.9, 137.2, 135.1, 133.8, 129.8, 129.3, 128.4, 128.3, 127.9, 126.8, 126.5, 126.0, 125.6, 122.0, 118.3, 118.2, 116.0, 115.7, 100.8, 60.9, 60.8, 35.6, 28.9, 28.4. HRMS calcd. For C_33_H_27_FINNaO_2_^+^ (M + Na^+^, *m*/*z*): 638.09627; found: 638.09727.

## 4. Conclusions

An interesting application of DOS of biologically promising compounds has been developed. A key feature was the application of the reagent-based diversification approach using a linker with different possibilities of cleavage. Starting from an immobilized primary library, diversity was expanded through many alternatives according to the reaction conditions used. A series of cholesterol absorption inhibitor analogues was obtained, as well as interesting hybrid compounds through incorporation of a chalcone group via cross-metathesis. Here, we have demonstrated the reliability of the methodology which can be suitable for generating large libraries of analogue structures.

## Figures and Tables

**Table 1:**
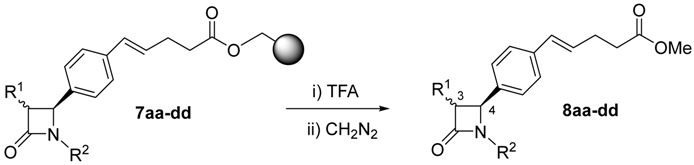
The secondary “trifluoroacetic acid (TFA)” library of β-lactams.

Entry	Starting Material	3,4 Configuration	R^1^	R^2^	Product	Yield (%) ^a^
1	**7aa**	*cis*	PhO	Bn	**8aa**	58
2	**7ab**	*cis*	PhO	Ph	**8ab**	43
3	**7bb**	*trans*	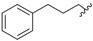	Ph	**8bb**	28
4	**7bc**	*trans*	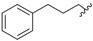	4-FPh	**8bc**	38
5	**7bd**	*trans*	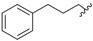	4-MeOPh	**8bd**	53
6	**7cb**	*trans*	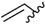	Ph	**8cb**	26
7	**7cc**	*trans*	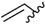	4-FPh	**8cc**	42
8	**7cd**	*trans*	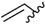	4-MeOPh	**8cd**	60
9	**7db**	*trans*	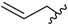	Ph	**8db**	55
10	**7dc**	*trans*	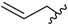	4-FPh	**8dc**	52
11	**7dd**	*trans*	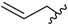	4-MeOPh	**8dd**	33

^a^ Overall isolated yield after flash column chromatography [five steps, based on loading of resin **2** (0.40 mmol/g, determined by elemental analysis of chlorine)].

**Table 2:**
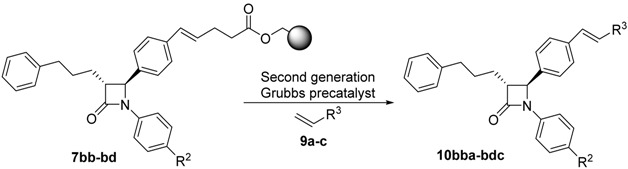
The secondary “Ru1” library of β-lactams.

Entry	Starting Material	R^2^	R^3^	Product	Yield (%) ^a^	CM Yield (%) ^b^
1	**7bb**	–H	MeO_2_C– ^c^	**10bba**	25	87
2	**7bd**	–OMe	MeO_2_C– ^c^	**10bda**	47	88
3	**7bd**	–OMe	Bn	**10bdb**	49	92
4	**7bc**	–F	Bn	**10bcb**	31	81
5	**7bc**	–F	2-BrPh	**10bcc**	27	50
6	**7bd**	–OMe	2-BrPh	**10bdc**	16	41

^a^ Overall isolated yield after flash column chromatography [four steps, based on loading of resin **2** (0.40 mmol/g, determined by elemental analysis of chlorine)]. ^b^ Yield of the cross-metathesis step calculated from the ratio between product yield and the yield of the corresponding β-lactams **8bb**–**bd** (see Table 1). ^c^ Product was esterified with diazomethane prior to purification.

**Table 3:**
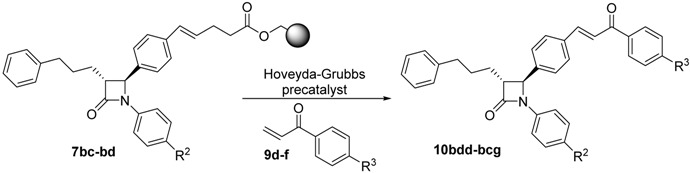
The secondary “Ru2” hybrid β–lactam library.

Entry	Starting Material	R^2^	R^3^	Product	Yield (%) ^a^	CM Yield (%) ^b^
1	**7bd**	–OMe	–CH_3_	**10bdd**	20	38
2	**7bd**	–OMe	–OCH_3_	**10bde**	15	28
3	**7bc**	–F	–I	**10bcf**	22	58
4	**7bc**	–F	–CH_2_Ph	**10bcg**	20 ^c^	53

^a^ Overall isolated yield after flash column chromatography [four steps, based on loading of resin **2** (0.40 mmol/g, determined by elemental analysis of chlorine)]. ^b^ Yield of the cross-metathesis step calculated from the ratio between product yield and the yield of the corresponding β-lactams **8bc**–**bd** (see Table 1). ^c^ Product could not be completely purified because of impurities.
